# Insights from model organisms on the functions of the tumor suppressor protein LKB1: Zebrafish chips in

**DOI:** 10.18632/aging.100319

**Published:** 2011-04-26

**Authors:** Yme U. van der Velden, Anna-Pavlina G. Haramis

**Affiliations:** Department of Molecular Genetics, Netherlands Cancer Institute, Amsterdam, The Netherlands

**Keywords:** LKB1, energy metabolism, cell polarity, animal models

## Abstract

The tumor suppressor LKB1 has emerged as a critical regulator of cell polarity and energy-metabolism. Studies in diverse model organisms continue to unravel the pathways downstream of LKB1; the emerging picture is that the outcomes of LKB1 signaling are mediated by a plethora of tissue-specific and context-dependent effectors.

## INTRODUCTION

In 1998, the gene responsible for the rare dominantly inherited disorder Peutz-Jeghers syndrome[1], characterized by gastrointestinal hamartomatous polyposis and an increased predisposition to cancer [2], was identified as *LKB1*, which encodes a serine/threonine protein kinase. In addition to the familial syndrome, somatic mutations in *LKB1* were later found in over 30% of lung adenocarcinomas [3] and as the first identified recurrent mutation in endometrial cancer[4]. However clues as to its function were first discovered only in 2003, when it was identified as the long sought-after kinase that activates the alpha subunit of AMP-activated protein kinase (AMPK) [5,6], linking LKB1 signaling to energy-metabolism control. Since then, LKB1 has been found to phosphorylate 12 other AMPK-related kinases including the microtubule-affinity-regulating kinase (MARK1-4), brain specific kinase (BRSK1-2), nuclear AMPK-related kinase (NUAK1-2), salt-inducible kinase (SIK1-3) and SNF-related kinase (SNRK) [7,8]. These results suggest that LKB1 is an upstream “master regulator”of energy homeostasis, cell polarity, DNA damage and cell cycle control [9].

Genetic analyses of LKB1 deficiency in higher eukaryotes have provided a framework to further dissect the functions of LKB1. However, the biology of LKB1 signaling appears to be highly complex, as loss of LKB1 function in invertebrates and vertebrates have generated divergent results in different tissues and contexts. In this research perspective, we review the current understanding of LKB1 function in cellular polarity and energy metabolism derived from loss-of-function studies performed in different model organisms. For a more comprehensive overview of LKB1, we refer the reader to these recent excellent reviews [10-15]. In the last section we will discuss how the recently generated Lkb1-deficient zebrafish can provide a new tool to gain important insight into the function of this tumor suppressor protein.

### LKB1 function during early development: polarization of the oocyte

Ten years before the human *LKB1* gene was cloned, the *C. elegans* homolog, abnormal embryonic PARtitioning of cytoplasm family member 4 (par-4), was retrieved from a maternal-effect-lethal screen for genes required for proper segregation of cytoplasmic factors in the first cell cycles of embryogenesis [16]. *par-4* mutant embryos had defects in several aspects of cell polarity and asymmetric cell division, which resulted in the formation of an amorphous mass of cells without distinct morphogenesis [17].

This function for LKB1 in polarization during early embryogenesis was subsequently found to be conserved, at least In *Drosophila melanogaster*. The *Drosophila* egg is a highly polarized structure well before fertilization and the origin of this polarization might even be traced back to the first cell division of the cytoblast in the fly ovary [18]. Differentiation of germline cells into oocytes coincides with asymmetric localization of proteins and mRNAs that set up the anterior-posterior (A-P) and dorsal-ventral (D-V) axes within the oocyte [18]. *lkb1* mutant germline clones showed disrupted localization of various mRNAs resulting in defective oocyte polarity [19]. LKB1-deficiency in follicle cells also led to polarization defects including disorganization of the epithelial monolayer [19]. These polarity defects were not fully penetrant and it was suggested that LKB1 is essential for the establishment of epithelial polarity in the follicle, but not for its maintenance [20]. However, under conditions of glucose starvation, polarity defects were observed in all examined follicle cells, indicating that LKB1 is critical also for the maintenance of epithelial polarity in follicle cells upon energetic stress. As LKB1 is known to regulate energy homeostasis, as outlined in more detail below, this suggests that diverse LKB1 functions are connected under certain physiological conditions. Similar results were obtained for *ampka* mutant follicle cells [20]. Indeed, many aspects of the polarity defects in LKB1-deficient follicle cells were rescued by introduction of a phosphomimetic *ampka* mutant demonstrating the involvement of the LKB1-AMPK axis in polarization during early development [21].

Together, this illustrates the high conservation of LKB1 function in the earliest polarization processes in both worms and flies. Although it remains to be determined whether this function is also conserved in vertebrates, interestingly LKB1 is asymmetrically localized to the animal pole in the mouse oocyte [22].

### LKB1 and cell polarization in later stages of development

LKB1 also has a conserved role in polarization during later stages of development. For example, loss of LKB1 signaling leads to impaired neuronal polarity in both invertebrates and vertebrates. In *C. elegans*, temperature-sensitive *par-4* mutants showed neuronal polarity defects in ventral cord neurons. This function was thought to be regulated by PAR-4-dependent phosphorylation of PAR-1, which is the homolog of human MAP/microtubule affinity-regulating kinases, MARK [23]. In *Drosophila*, depletion of LKB1 in neuroblasts caused polyploidism in larval brains, but via a Par1-independent mechanism [24]. Instead, defects in mitotic spindle formation and mislocalization of the Baz/PAR-6/aPKC complex, a protein complex involved in cellular polarity, likely contributed to the reported phenotype [24]. In mice, conditional *lkb1* deletion in telencephalic progenitors led to impaired polarization of cortical neurons through impaired activation of the AMPK-related kinases SAD-A/B. Thus, LKB1 is required for polarization also in the vertebrate brain, although the molecular mechanisms involved are to a certain extent organism-specific [25].

In addition to neuronal polarization, LKB1 has been implicated in the polarization of epithelial structures, such as photoreceptors in the *Drosophila* eye. The *Drosophila* retina is derived from the eye imaginal disc, which is an epithelial structure. Eye-specific inactivation of LKB1 led to severe loss of polarity in photoreceptors at pupal stages [26]. Importantly, AMPK was not the primary LKB1 target in *Drosophila* eye development, but rather other AMPK-related kinases including SIK, NUAK and PAR-1 [26]. In vertebrates, activation of LKB1 induced complete polarization of single intestinal epithelial cells in culture [27]. Furthermore, AMPK activation is required for tight-junction formation and polarization in the Madin-Darby Canine Kidney (MDCK) epithelial *cell* line, although this may not be exclusively dependent on LKB1 [28,29]. LKB1 null mice do not survive beyond E10.5 and show several defects including mesenchymal cell death as well as neural tube and vascular abnormalities associated with increased VEGF signaling [30]. Somewhat unexpectedly, inactivation of *Lkb1* in several mouse tissues did not lead to gross epithelial polarity defects, with the notable exception of the pancreas [31,32].

Thus, it appears that LKB1 regulates polarization during development throughout the animal kingdom in a tissue- and context-dependent manner, and via phosphorylation of distinct substrates.

### LKB1: a master regulator of energy homeostasi

Probably the best-studied function of LKB1 to date, at least in vertebrates, is the regulation of energy homeostasis, particularly through AMPK activation and the target of rapamycin (TOR) pathway [33,34]. Upon energetic stress induced by a variety of stimuli such as food-deprivation, exercise, osmotic stress and hypoxia, AMPK is phosphorylated and activated by LKB1. AMPK then phosphorylates tuberous sclerosis complex 2 (TSC2), which leads to inhibition of TOR complex 1 (TORC1) activity [34]. TORC1 activity is associated with cell growth and viability since TORC1 stimulates anabolic processes such as protein synthesis while inhibiting catabolic processes like the degradation of cellular components by autophagy [35]. Thus, upon LKB1-dependent activation of AMPK, TORC1 signaling is inhibited, which promotes energy conservation under conditions of energetic stress.

Given the embryonic lethal phenotype of the knockout mouse, the role of LKB1 in energy homeostasis at the whole organism level in animals has only been studied in *C. elegans* and, more recently, in *D. rerio*. These studies, which are described in detail below, have revealed a far more complex role for LKB1 in energy homeostasis beyond only the regulation of TOR signaling via AMPK. Indeed, in addition to TSC2, AMPK has a multitude of direct substrates, many of which are also involved in metabolism control [11,14]. In *C. elegans*, larvae developmentally arrest and enter the so-called “dauer” phase under unfavorable environmental conditions. Dauer larvae do not feed, become stress-resistant, are extremely long-lived and “non-aging” [36]. In order to ensure long-term survival, fat is stored in the hypodermis, which is an organ akin to the skin of higher organisms [37]. Dauer larvae with compromised LKB1/AMPK signaling rapidly depleted hypodermic fat storages and die prematurely due to vital organ failure [38]. This inappropriate fat depletion was found to be due to increased activity of adipose triglyceride lipase (ATGL-1), a direct target of AMPK. Similar to this result in *C. elegans*, we recently reported that *lkb1* mutant zebrafish are also unable to cope with energetic stress [39]. Although Lkb1 deficiency in *D. rerio* did not lead to overt developmental defects, *lkb1* mutants did fail to downregulate metabolism once the yolk, which provides energy in the first days of development, was consumed. These *lkb1* mutants exhibited hallmarks of a starvation response at the cellular and biochemical level, displayed profoundly decreased ATP levels and became energy-depleted much sooner that food-deprived wild type animals. Thus, in both worms and zebrafish, LKB1 is essential for control of whole-body energy homeostasis and adaptation of metabolism to changes in energy availability, which is essential for long-term viability of the organism.

Zebrafish *lkb1* mutants die two days after yolk absorption in stark contrast to wild-type larvae that can survive food deprivation for more than six days. Interestingly, two days of food deprivation did not lead to detectable AMPK phosphorylation in wild-type larvae, suggesting that deregulated AMPK signaling may not be the sole cause for impaired energy metabolism control in *lkb1* larvae. Furthermore, TOR signaling was not severely deregulated in *lkb1* mutants. Thus, we proposed that the AMPK-TORC1 axis might not be the critical or only effector of Lkb1-mediated maintenance of whole-organism energy homeostasis, at least in this setting. Interestingly, recent work on the effect of *Lkb1* inactivation in mouse hematopoietic stem cells showed that, while LKB1 was critically required to regulate energy metabolism and maintain cell survival, the effects were again largely independent of AMPK and TORC1 signaling [40-42]. Together, these findings illustrate that *in vivo* LKB1 controls metabolism though several pathways in addition to TORC1 signaling and showcase the complexity of LKB1 biology.

### A zebrafish perspective on LKB1

As zebrafish *lkb1* mutants survive embryonic development, unlike mice, they provide the first embryonic viable vertebrate model of homozygous *lkb1* deletion. This, combined with the many advantages of using zebrafish as a model organism, some of which are described below, should rapidly advance our understanding of LKB1 function.

One of the advantages of zebrafish is that the oocyte is externally fertilized, allowing early developmental processes, from fertilization onwards, to be easily analyzed. In addition, germline replacement methods [43] mean that an animal lacking both maternal and zygotic LKB1 can be generated. Maternal-zygotic zebrafish *lkb1* mutants will provide a system to address whether and how Lkb1 functions in the first cleavage stages in vertebrates.

Another attractive feature of the zebrafish is their small size and transparency during development. In combination with the availability of numerous transgenic lines expressing tissue-specific fluorescently-labelled reporters, this allows real-time, *in vivo* visualization of various processes such as cell migration and organogenesis. Thus, questions pertaining to the biology of tissue physiology in a setting of Lkb1-deficiency can be addressed.

Although addressing whether neuronal polarity was impaired was beyond the scope of our previous study, it is still possible that Lkb1 is required for polarization or asymmetric cell division in neuronal tissues in zebrafish, given this function is conserved in *C. elegans* and *Drosophila*. Should that be the case, the ease of performing forward genetic screens in zebrafish could help to dissect the pathway of neuronal polarization in vertebrates by identification of new proteins involved in this process.

Interestingly, we did not observe polarity defects in either the gut or the eye of zebrafish *lkb1* mutants, in contrast to studies in human cell lines and *Drosophila* respectively, again highlighting the cell-type specificity and context-dependency of LKB1 function.

Since Lkb1 deficiency leads to impaired metabolic control upon energetic stress, it will be interesting to determine whether *lkb1* mutants are hypersensitive to other types of stress, such as osmotic stress and DNA damage. Our preliminary results showed that *lkb1* mutants are hypersensitive to mechanical stress, but only when they are under energetic stress, again illustrating that the metabolic functions of Lkb1 are tightly linked with other Lkb1-dependent processes.

**Figure 1.**
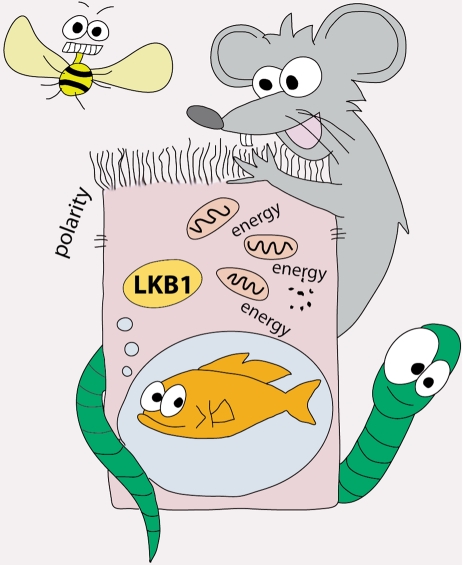
Studies of LKB1-deficiency in flies, worms, mice and zebrafish have revealed that the tumor suppressor LKB1 has conserved and divergent roles in the regulation of cell polarization and energy metabolism processes

Finally, since zebrafish *lkb1* mutants are embryonic viable they provide an excellent platform to conduct chemical genetic screens to identify molecular pathways that are regulated and/or cooperate with Lkb1 and lead to deregulation of metabolism. These types of screens could also identify compounds that can modulate metabolism and may prove to be useful for inhibiting growth of LKB1-deficient tumors.

## CONCLUSION

*LKB1* is a tumor suppressor gene and is mutated in a wide variety of human cancers. Thus, deciphering its function could have direct clinical implications. Given the complexity of LKB1 function, which is illustrated by the diversity of its mutant phenotypes in a variety of model organisms and contexts, *lkb1* mutant zebrafish offer a powerful new tool for unraveling the numerous mechanisms and pathways regulated by LKB1. It also provides the unique opportunity to study LKB1 function at the whole organism level in vertebrates.
